# Machine learning-assisted lens-loaded cavity response optimization for improved direction-of-arrival estimation

**DOI:** 10.1038/s41598-022-12011-z

**Published:** 2022-05-20

**Authors:** Muhammad Ali Babar Abbasi, Mobayode O. Akinsolu, Bo Liu, Okan Yurduseven, Vincent F. Fusco, Muhammad Ali Imran

**Affiliations:** 1 Institute of Electronics, Communications and Information Technology (ECIT), Queen’s University Belfast, Belfast, UK; 2 Faculty of Arts, Science and Technology, Wrexham Glyndŵr University, Wrexham, LL11 2AW UK; 3 James Watt School of Engineering, University of Glasgow, Glasgow, UK

**Keywords:** Applied physics, Techniques and instrumentation, Electrical and electronic engineering

## Abstract

This paper presents a millimeter-wave direction of arrival estimation (DoA) technique powered by dynamic aperture optimization. The frequency-diverse medium in this work is a lens-loaded oversized mmWave cavity that hosts quasi-random wave-chaotic radiation modes. The presence of the lens is shown to confine the radiation within the field of view and improve the gain of each radiation mode; hence, enhancing the accuracy of the DoA estimation. It is also shown, for the first time, that a lens loaded-cavity can be transformed into a lens-loaded dynamic aperture by introducing a mechanically controlled mode-mixing mechanism inside the cavity. This work also proposes a way of optimizing this lens-loaded dynamic aperture by exploiting the mode mixing mechanism governed by a machine learning-assisted evolutionary algorithm. The concept is verified by a series of extensive simulations of the dynamic aperture states obtained via the machine learning-assisted evolutionary optimization technique. The simulation results show a 25$\%$ improvement in the conditioning for the DoA estimation using the proposed technique.

## Introduction

Accurate direction-of-arrival (DoA) information is a key requisite for mmWave channel sounding. Classical methods of DoA estimation require an array of antennas connected to the associated radio frequency (RF) hardware per antenna, also called the RF-chain, in conjunction with techniques such as the ESPRIT^1^, Capon^2^, Bartlett^3^ and MUSIC^4,5^ algorithms. The development of antenna arrays and RF chains can be both complex and costly at mmWave frequencies. This is because an increase in the number of antennas required to provide sufficient angular discrimination compensating for the high path loss at mmWave frequencies can aggressively enhance the complexity and cost of the mmWave radio hardware. Moreover, multiple RF-chain systems need a high degree of hardware thermal considerations, adding further complexity to the system^6,7^. As an alternative, recently a number of mmWave antenna hardware simplification approaches that focus on classical beam synthesis approaches using fewer RF chains have been investigated^3,8–11^. Highly directional frequency-diverse antenna apertures have also been investigated as a promising alternative to a fully connected antenna array and RF-chain system. Frequency-diverse antenna aperture is derived from microwave computational imaging concepts (e.g.^12–16^), where field-of-view (FoV) information is captured and reconstructed, using single or sometimes multiple RF chains^17^. Recently, it has been shown that channel information within an FoV (in terms of far-field radiation) can also be constructed from quasi-random measurement modes using computational techniques^18^. A preliminary theoretical investigation of DoA estimation using a mode-mixing cavity was presented in^18^. However, this was limited by the use of a hypothetical frequency-diverse antenna aperture with high-Q factor. A numerical and experimental validation of DoA estimation using a lens-loaded cavity aperture was presented in^19^. These works (i.e.^18,19^) both adopted computational methods for the DoA estimation, carried forward in this paper.

In this work, it is shown that DoA estimation capabilities of a lens-loaded cavity can be systematically enhanced by converting it into a lens-loaded dynamic aperture optimized efficiently. This is implemented by introducing dynamic reconfigurability into the lens-loaded cavity by adding a mechanically controlled mode-mixing mechanism; thus, adding another dimension to physically control the aperture performance. The benefit of using a lens structure placed in front of the cavity is that it enhances the quasi-random variations in the radiation modes previously shown in^19,20^; hence, impacting positively to the spatio-temporal bases, which in turn improves the DoA estimation accuracy. This is then followed by dynamically reconfiguring the lens-loaded aperture optimized by a machine learning (ML)-assisted evolutionary algorithm for a given wireless channel, which enhances the DoA estimator performance further, shown in this paper. To circumvent the need for a reasonably good initial design, ad-hoc process, and a large number of full-wave electromagnetic (EM) simulations which are often needed by popular global optimization techniques (e.g., evolutionary algorithms), an ML-assisted antenna design optimization algorithm from the surrogate model-assisted differential evolution for antenna synthesis (SADEA) series^21–25^ is employed for the targeted aperture optimization. In comparison to standard global optimization methods (e.g., particle swarm optimization), the selected algorithm (i.e., SADEA-I^21^) provides up to 20 times speed improvement, while obtaining design solutions of comparable or better quality for many antenna cases^26^, making it a good choice for the targeted problem. SADEA-I employs the surrogate model-aware evolutionary search (SMAS) framework for surrogate model management^27^, which shows a harmonious balance between evolutionary algorithm-based global search and surrogate modeling.

The key contributions of this work are summarized as follows:

- A novel lens-loaded dynamic aperture geometry with an associated computational DoA estimation system is proposed with the capability of updating itself.
- It is shown for the first time that mechanical rotation of a mode-mixing scatterer updates the state of a frequency-diverse antenna, resulting in a unique set of radiation modes.
- It is shown for the first time that ML-assisted antenna design optimization techniques (in our case, SADEA-I) are well suited for simulation-driven lens-loaded dynamic aperture optimization.

**Figure 1:**
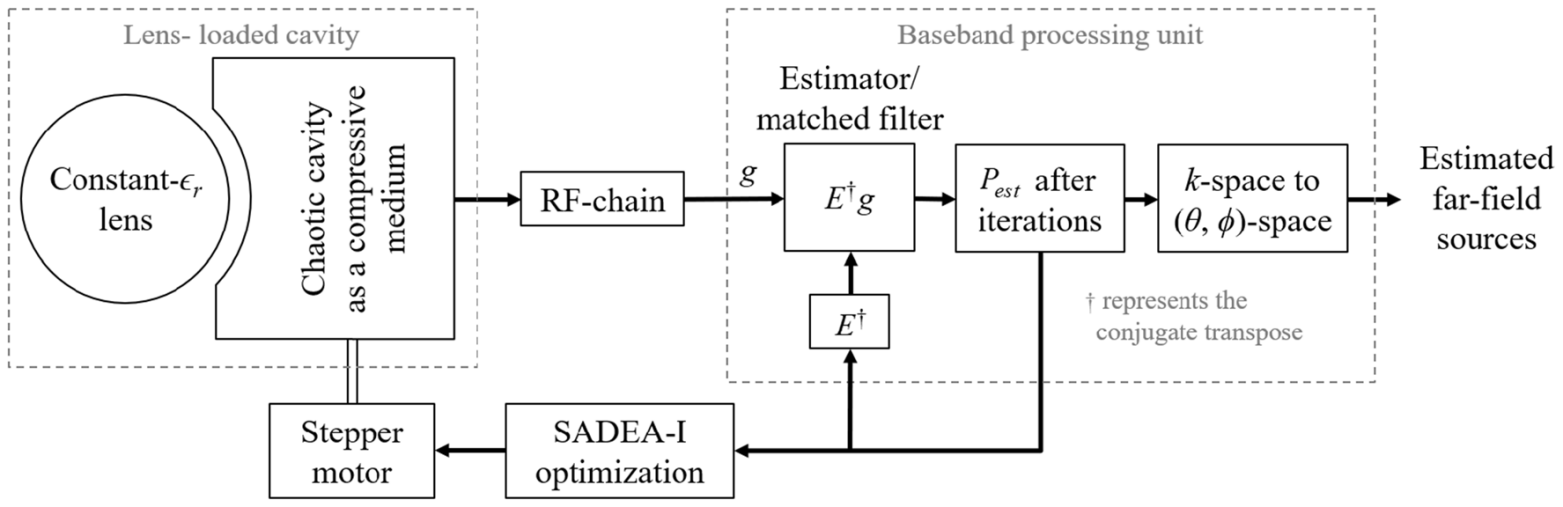
Operational block diagram of a state-diverse mode lens-loaded cavity aperture.

## System model and methods

### Lens-loaded dynamic aperture

**Figure 2:**
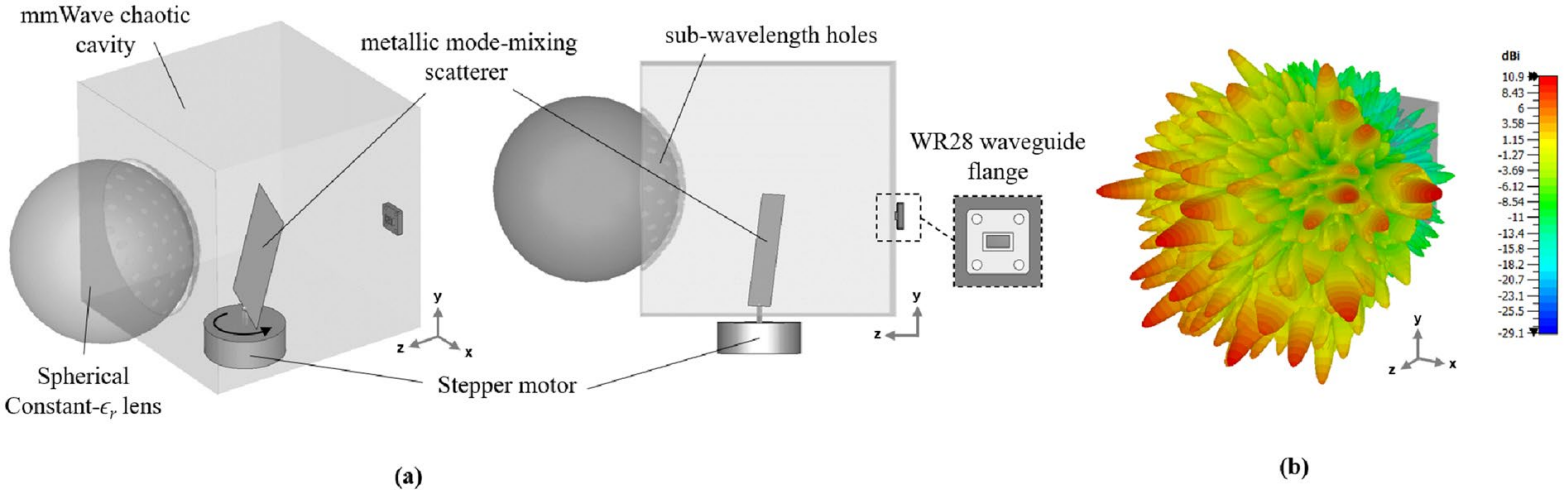
(**a**) Lens-loaded cavity structure with a mode mixing scatterer connected to a stepper motor to include state-diversity. (**b**) Simulated peak realized gain representing high and low magnitude values on the radiation mask when a test signal of 28.1 GHz is excited at the WR28 input.

The system block diagram is shown in Fig. 1—a single-input single-output lens-loaded cavity antenna is connected to a baseband processing unit via a single RF chain. The lens-loaded cavity antenna in and of itself serves as a replacement for an antenna array aperture, so therefore, it is simply referred to as an aperture in this work. The processing unit comprises an estimator or a matched filter responsible for the DoA estimation. The lens-loaded cavity comprises an oversized chaotic cavity operating as a frequency-diverse compressive medium^18^. A constant-$\varepsilon _r$ lens is placed in front of the chaotic cavity while the medium of EM energy transfer between the cavity and the lens structure is a curved surface with sub-wavelength holes. The structural configuration of the lens-loaded cavity is clearer in Fig. 2a where the perspective view shows the surface of the sub-wavelength hole. The lens-loaded cavity is placed in a Cartesian coordinate system where its FoV is along the $+z$-axis. When looked at from one side, i.e., *xy*-plane, a portion of the lens can be seen to be submerged in the chaotic cavity, however, it is important to note that the lens is making any contact with the cavity. The gap between the lens and the surface containing the sub-wavelength holes is governed by the focal length of the lens at the frequency of operation, which is 28 GHz for this particular case. Note that the test frequency band in this work is among the mmWave 5G as defined by the 3GPP New Radio (NR) FR2 enlisting n257. Details of the synthesis approach to developing a constant-$\varepsilon _r$ lens for a given frequency can be found in^28^ and the works discussed therein. The spherical constant-$\varepsilon _r$ lens (see Fig. 2a) used in this study has a radius of 66.5 mm while the distance between the centre of the lens and the chaotic cavity is 70 mm. A careful adjustment of this gap is critical for the best radiation performance of the lens-loaded cavity. As an example, the radiation performance of the cavity in terms of simulated peak realized gain is shown in Fig. 2b.

The chaotic cavity in Fig. 2a has physical dimensions of 170 mm $\times$ 178 mm $\times 180\,{\text {mm}}$ in (*x* $\times$ *y* $\times$ *z*) directions. The cavity structure is basically a metallic box with a simple geometric configuration. The constant-$\varepsilon _r$ lens is placed in front of the cavity (i.e., facing the +*z*-axis) while RF chain is connected to the back side (facing $-z$-axis). For 28 GHz operation, a standard waveguide probe (WR28) is used to connect the cavity to the RF chain and the subsequent computation DoA estimation system. The most important component of the chaotic cavity relevant to this study is the metallic mode mixing scatterer. The scatterer is a metallic strip of size 78 mm $\times$ 45 mm randomly oriented and asymmetrically placed within the chaotic cavity. The main purpose of the scatterer is to enhance the quasi-randomness of the cavity by randomly reflecting the EM energy within the metallic structure. This is similar to other mode-mixing structures in^13,14^ and the works discussed therein. However, the unique feature of the mode-mixing scatterer of this kind is that with a slight rotation, the disturbance in the wave-chaotic medium results in a new set of radiation modes. This feature allows to dynamically reconfigure the chaotic cavity by simply controlling the rotation of the mode-mixing scatterer by connecting it to a stepper motor, as achieved in this study. This reveals that by controlling a single parameter (i.e., angle of rotation of the mode-mixing scatterer) of the aperture, it is possible to generate unique sets of radiation modes; hence, determining the best angle of rotation can be formulated as a 1-D optimization problem that can be solved in a short time.

Even though the problem definition above bears a semblance of a partial geometry modification problem, it is not a typification of partial geometry modification problems in which parts of the EM structure are removed or replaced for alteration and modification^29,30^. This is mainly because the geometry of the EM structure (and its associated elements) in this study, in and of itself, is not altered or modified. Rather, the EM structure (unaltered and unmodified in terms of physical geometry) is characterized, as the mode-mixing scatterer (also with a consistent geometry and connected to a stepper motor) is rotated for various angular states to establish the near-optimum state for DoA estimation. The connection settings are shown in Fig. 2a. It is worth mentioning that it does not really matter to which side of the chaotic cavity the mode-mixing scatterer is attached via the stepper motor shaft; however, it is recommended that the mode-mixing scatterer is placed close to a corner of the chaotic cavity to ensure that the physical symmetry of the structure along all three axes is broken. For this purpose, the scatterer in this study is placed at positions (87 mm, 42 mm, 65 mm) along (*x*, *y*, *z*) from the three walls of the chaotic cavity. Another point to remember here is that the scatterer needs to be firmly fixed to the stepper motor to ensure the chaotic cavity retains its physical state at any particular rotation angle. Also, note that a rotation mechanism with enhanced rotational resolution can lead to quasi-continuous control of the scatterer.

### Dynamic aperture optimization methodology

Considering the targeted simulation-driven aperture design optimization problem, there are several local and global optimization methods in the literature, such as^26,31–33^. Local optimization techniques rely on good initial designs that the designer needs to specify as starting points^31^. However, in our case, it is difficult to find a good initial design. Global optimization-based EM device design techniques (e.g.^33–35^) do not require initial designs, but they often require a large (sometimes prohibitive) number of EM simulations to obtain optimal results^26,33–35^. For our targeted aperture, each EM simulation costs more than one hour. Hence, both kinds of methods are not suitable.

In recent years, the incorporation of ML techniques into the optimization kernel of standard EAs has been demonstrated to lower the computational cost of the optimization process, which is applied to EM device design^36–38^. This is mainly achieved through surrogate model-based optimization in which many computationally expensive EM simulations in the optimization process are replaced with surrogate model-based predictions. These surrogate models, also called metamodels, are computationally cheap approximation models of expensive full-wave EM simulations. They are often constructed using ML techniques and are used to emulate the characterization or behavior of the EM simulation model, as closely as possible. Even though many paradigms and methods are currently available for the ML-assisted optimization of EM designs as reported in^36–39^, some of these approaches still have the drawbacks of standard optimization methods and are not general due to the ad-hoc processes required to ensure their efficiencies.

The approaches in^40–42^ require good initial designs or starting points and may get trapped in local optima due to their use of a local search mechanism, trust-region gradient search. In^43–45^, the fidelity of the EM model is varied methodically in the optimization process to improve efficiency. This is implemented alongside ad-hoc processes such as verification and improvement of the generated designs using high fidelity simulations and input space mapping in the local region, respectively, and the use of user-defined thresholds to control the variance of the fidelity of the EM model in terms of cells or lines per wavelength. These methods are not applicable for our case because: (1) a good initial design cannot be deduced for the lens-loaded aperture a priori, as earlier mentioned, (2) the discretization of lens-loaded aperture requires millions of mesh cells at the host of a relatively long simulation time (even for a relatively low mesh density, see section “Lens-loaded dynamic aperture operation”) to guarantee model accuracy. So, having an accurate coarse (low fidelity) model with a low cost in terms of simulation time is not feasible. SADEA-I^21^, adopted in this work, helps to overcome these drawbacks by providing a methodology that implements supervised learning and evolutionary computation in a unified optimization framework to efficiently synthesize the lens-loaded aperture for mmWave DoA estimation. The supervised learning and evolutionary computation techniques and their harmonized framework in SADEA-I are discussed as follows:

#### Supervised Learning

Like other methods in the SADEA series^22–25^, SADEA-I uses Gaussian process (GP)^46,47^ for surrogate modelling. Given a set of EM design geometric and/or material properties ($x=(x^{1},\ldots ,x^{n})$), corresponding to a set of performances ($y=(y^{1},\ldots ,y^{n})$) from full-wave EM simulation results, GP predicts the targeted EM design performances ($y=f(x)$) for a candidate design *x* by modelling *y*(*x*) as a Gaussian distributed stochastic variable having a mean of $\mu$ and a variance of $\sigma _1^2$. If *y*(*x*) is continuous, as it is the case for typical EM device design landscapes, the function values ($y(x^i)$ and $y(x^j)$) of any two candidate designs such as $x^{i}$ and $x^{j}$ should be in proximity if they are highly correlated. A Gaussian correlation function is used to deduce this correlation between two candidate designs in SADEA-I:

1 $$\begin{aligned} \begin{array}{ll} Corr(x_{i},x_{j})=e^H; \quad H=-\sum \limits _{t=1}^{d}\zeta _{l}|x_{i}^{t}-x_{j}^{t}|^{\rho _{t}} \\ \text {for} \quad \zeta _{t}>0, 1\le \rho _{t}\le 2 \\ \end{array} \end{aligned}$$

where *d* is the dimension of *x* and $\zeta _{l}$ is the correlation parameter that determines how rapidly the correlation diminishes as $x_{i}$ moves in the *t* direction. The smoothness of the function is related to $\rho _{t}$ with respect to $x^{t}$. To deduce the parameters $\zeta _{t}$ and $\rho _{t}$, the likelihood function that $y=y^{i}$ at $x=x^{i} (i=1,\ldots ,n)$ is maximized. Hence, the Gaussian process regression or kriging-based prediction of the performance ($y(x^{*})$) of a candidate design ($x^{*}$) is carried out as follows:

2 $$\begin{aligned} {\hat{y}}(x^{*})={\hat{\mu }}+z^{T}Z^{-1}(y-I{\hat{\mu }}) \end{aligned}$$

where

3 $$\begin{aligned} Z_{i,j}= & {} Corr(x_{i},x_{j}), i,j=1,2,\ldots ,n \end{aligned}$$

4 $$\begin{aligned} z= & {} [Corr(x^{*},x_{1}),Corr(x^{*},x_{2}),\ldots ,Corr(x^{*},x_{n})] \end{aligned}$$

5 $$\begin{aligned} {\hat{\mu }}= & {} (I^{T}Z^{-1}I)^{-1}I^{T}Z^{-1}y \end{aligned}$$

The mean squared error of the prediction uncertainty is:

6 $$\begin{aligned} {\hat{s}}^2(x) = \hat{\sigma _1}^{2}[I-z^{T}Z^{-1}z+(I-z^{T}Z^{-1}z)^{2}(I^{T}Z^{-1}I)^{-1}] \end{aligned}$$

where

7 $$\begin{aligned} \hat{\sigma _1^{2}}=(y-I{\hat{\mu }})^{T}Z^{-1}(y-I{\hat{\mu }})n^{-1} \end{aligned}$$

A number of prescreening methods are available for the appraisal of the quality of a candidate design with respect to the predicted value in Eq. (2) and the prediction uncertainty in Eq. (6)^48^. In SADEA-I, the lower confidence bound (LCB) method^49^ is used. If the predictive distribution of *y*(*x*) is $N({\hat{y}}(x), {\hat{s}}^2(x)$ for *y*(*x*), then the LCB prescreening of *y*(*x*) can be estimated as follows:

8 $$\begin{aligned} \begin{array}{ll} {\hat{y}}(x)-L {\hat{s}}(x) \\ L \in [0,3] \\ \end{array} \end{aligned}$$

where *L* is a constant that is often set to 2 to have a good balance between exploration and exploitation^48^.

#### Evolutionary computation

The EA driver in the SADEA-I is differential evolution (DE)^50^. DE is a popular EA widely used in engineering optimization. It outperforms many other EAs for continuous optimization problems^50^. Suppose that $P_{designs}$ is a population of candidate designs in the aperture optimization process. Let $x \in R$ be a candidate design (individual solution) in $P_{designs}$. To generate a child solution *C* for *x*, mutation is first carried out to produce a donor vector:

9 $$\begin{aligned} v^{i}=x^{best}+F \cdot (x^{r_2}-x^{r_3}) \end{aligned}$$

where $x^{best}$ is the best individual of the current population having a size of $P_{designs}$ by 1, and $x^{r_1}$ and $x^{r_2}$ are two mutually exclusive solutions randomly selected from $P_{designs}$; $v^{i}$ is the $i^{th}$ mutant vector in the population after mutation; $F\in (0,2]$ is the scaling factor (a control parameter). The mutation strategy in Eq. (9) is called DE/best/1. After the mutation is completed, the following crossover operator is applied to produce the child, *C*, as follows:

1. Randomly select a variable index $j_{rand} \in \{1, \ldots , P_{designs}\}$,
2. For each $j=1$ to $P_{designs}$, generate a uniformly distributed random number *rand* from (0, 1) and set:
  10 $$\begin{aligned} C_j = \left\{ \begin{array}{ll} v_j, &{}\quad \hbox { if (}\ r \, and\le CR) | j=j_{rand} \\ x_j, &{}\quad \text{ otherwise } \\ \end{array} \right. \end{aligned}$$
  where $CR\in [0,1]$ is the crossover rate (a constant).

Note that since the EA process is 1-D, the DE mutation and crossover operations (Eqs. (9) and (10) , respectively) are implemented using populations with a size of $P_{designs}$ by 1, as detailed above. Additional details on how DE mutation and cross over operations are implemented generally and specifically can be found in^50^.

#### The SADEA-I method

The essential steps of SADEA-I for the lens-loaded aperture optimization are described as follows^21^:

- **Step 1:** Using the Latin Hypercube sampling method^51^, a small number ($\alpha$) of designs are sampled from the design space of the lens-loaded aperture, and full-wave EM simulations are carried out to obtain their performances. The initial database is created using these designs and their simulation results.
- **Step 2:** If a preset stopping criterion such as the maximum number of EM simulations is met, output the best design from the database; otherwise go to Step 3.
- **Step 3:** Select the $\gamma$ best designs from the database to form a population of $P_{designs}$ having a size of $P_{designs}$ $\times$ 1, and update the best solution obtained so far.
- **Step 4:** Apply DE mutation and crossover operations (Eqs. (9) and (10) , respectively) on $P_{designs}$ (the size is as described in Step 3) to generate child populations having $\gamma$ child solutions each.
- **Step 5:** For every candidate design in each population, build a GP surrogate model using the nearest designs based on Euclidean distance from the database and their simulation results as the training data points.
- **Step 6:** Use the surrogate models in Step 5 to prescreen the child solutions in Step 4 according to Eq. (8), and select the best child solution based on the LCB values.
- **Step 7:** Evaluate (simulate) the prescreened best child solution from Step 6. Add it and its simulation results to the database. Go back to Step 2.

In terms of algorithm parameters (see section “Example and discussion”), $\alpha$ = 20, $\gamma$ = 20 and *F* = 0.8 are used.

## Lens-loaded dynamic aperture operation

**Figure 3:**
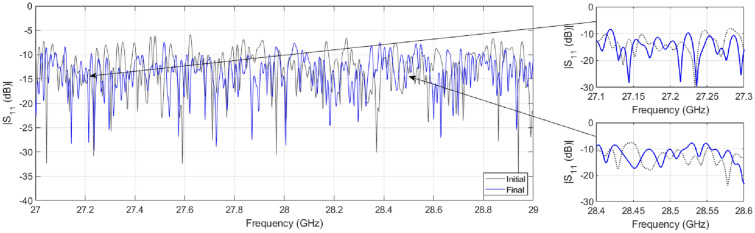
Comparison between the return loss before and after the mode optimization step.

**Figure 4:**
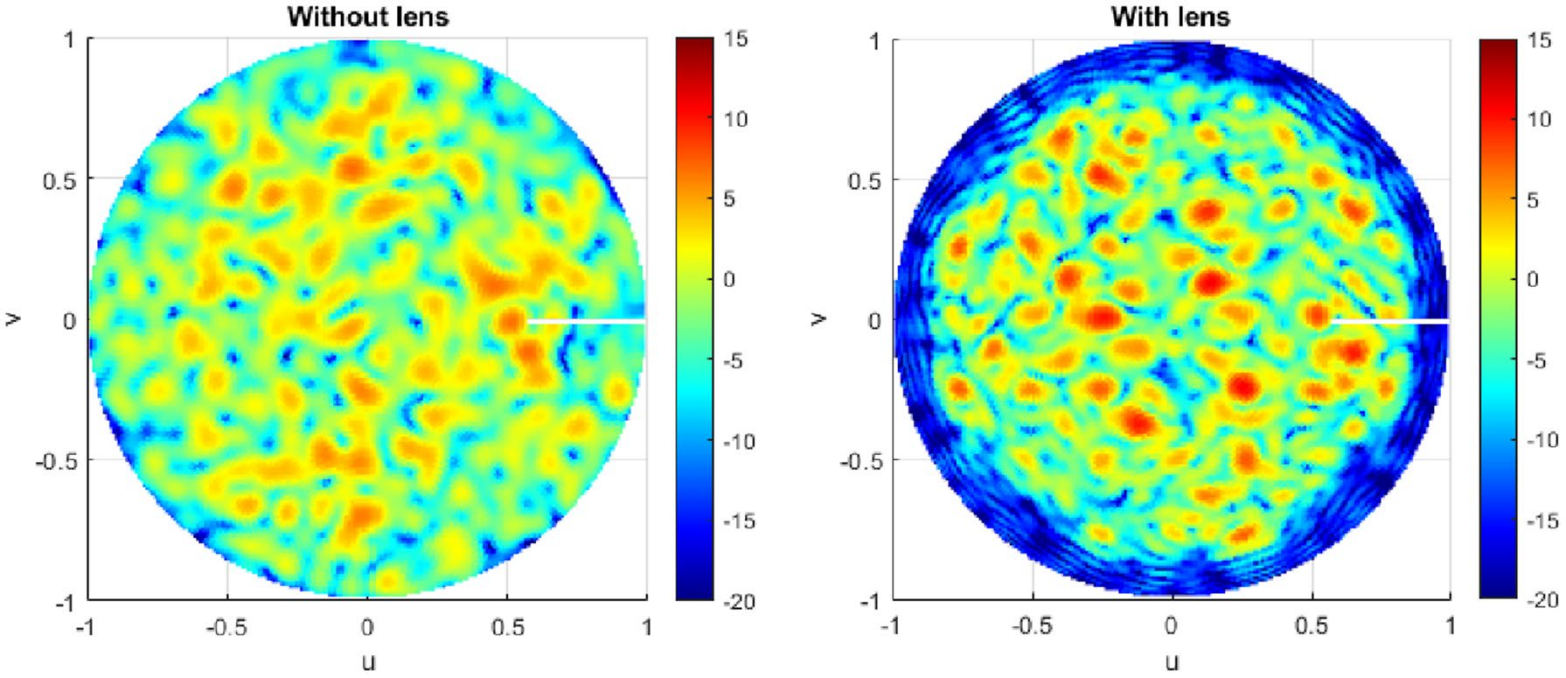
Impact of the presence of constant-$\varepsilon _r$ lens in front of the chaotic cavity on the FoV described using simulated peak realized gain at a test frequency of 28.1 GHz in *UV* plane (normalized).

To understand the proposed lens-loaded dynamic aperture optimization technique proposed in this work, it is important to first look at the block level operation of the system when the mechanical state of the metallic scatterer is fixed. In other words, when there is no input to the stepper motor (see Fig. 1) and SADEA-I-based optimization process is not yet initiated. The operation of the lens-loaded cavity in this state can be understood by looking at the radiation modes excited by the lens-loaded cavity shown in Fig. 2b. Here, the input of the lens-loaded cavity is excited by a 28 GHz signal, and the radiation in terms of far-field realized gain values is recorded at test frequencies within the range of 27–29 GHz. Full-wave EM simulations are carried out using the transient finite integration technique (FIT) solver in CST microwave studio with an accuracy of − 50 dB. As can be seen in Fig. 2b, the structure has quasi-random radiation with high and low gain values spread across the azimuth and elevation directions within the FoV along the *x*-axis. Note that the 3D plot of the realized gain magnitude (referred to as radiation mask from this point onward) will be unique (and different) if the input signal is changed from 28 to 28.05 GHz. This phenomenon and its benefit to the spatial incoherence of the radiation modes are discussed in the preceding investigation^19^. Conversely, if the lens-loaded cavity is used as a receiver and a broadband far-field source’s signal is impinging on the lens structure, the signal will use a similar wave-chaotic transfer function, *E*. This is evident in Fig. 3, where closely-spaced radiation modes can be seen, corroborating the benefits of multiple modes generation in a frequency diverse antenna^18^. The advantage of placing the lens in front of the chaotic cavity is that it confines the radiated energy within the FoV, depicted via the radiation mode mask shown in Fig. 4. As a result, the lens structure enhances the peak realized gain value of the radiation mask, making it as high as 6 dBi. This is because when the lens-loaded cavity is used as a receiver, the lens structure helps in delivering comparatively larger energy to the mode mixing cavity compared to when there is no lens placed in front of the cavity^15,19^. The operational FoV for the lens-loaded cavity spans across $\sim 120^\circ$ both along with azimuth and elevation plans directions^20^. Assuming that the field distribution radiated by different sources incident on the aperture is *P* defined as $P = e^{-jk_0(y\sin \theta \cos \phi - x\sin \theta \sin \phi )}$, when $k_0$ is the wave number). The compressed measurements, *g*, can be correlated to *P* through the aperture radiated fields projected on a characterization plane giving the transfer function, *E*, as follows^19,52,53^:

11 $$\begin{aligned} g(\omega )=\int _{\overline{r}}{E(\overline{r} ,\omega ,k)P(\overline{r})d\overline{r}+n(\omega )} \end{aligned}$$

In Eq. (11), *n* denotes the system noise, $\overline{r}$ refers to the coordinates across the aperture, $\omega$ is the frequency for frequency-diverse operation and *k* is the aperture state. From Eq. (11), an estimate of discretized *P*, $P_{est}$, can be deduced by means of a simple matched-filtering operation, $P_{est}=E^\dagger g$, where $^\dagger$ is the Hermitian transpose. Finally, the Fourier transform of $P_{est}$ produces the DoA estimation pattern. The exponential decay of the impinging signal on the lens-loaded cavity dictates the impulse response in time domain $h(t)=n(t)\exp ^{-\frac{2t}{\tau }}$, which is proportional to the Q-factor of the structure. Here, *n* is $N(0,\sigma _2^2)$ and *N* is normal distribution having a mean of 0 and variance of $\sigma _2^2$, and $\tau$ is the the centroid absolute value of the impulse response. Calculating the Q-factor of the lens-loaded cavity (Fig. 2) using $Q=\pi f_0 \tau$ gives a value of  4600, evaluated by studying *h*(*t*) using full-wave EM simulations. Given the Q-factor, the theoretical number of modes can be calculated using $M = QB/f_0$, which is $\sim 300$ in the static state of the lens-loaded cavity. It has been elaborated in^19^ that the DoA estimation is possible via this static state of the lens-loaded cavity structure by using the iterative method for least-squares reconstruction, governed by:

12 $$\begin{aligned} P_{est+1,M} = \arg \min \left\| g_{N} - E_{N \times M}P_{est,M} \right\| _{2}^{2}, \end{aligned}$$

where *N* is the number of modes and *M* is the number of pixels on the characterization plane while the match-filter solution $P_{est,M}=E^{\dagger }_{N \times M}g_N$ is used as an initial estimation. When the source projection patterns are estimated, the DoA estimation can be retrieved by performing a Fourier transformation operation on the final $P_{est}$. The final DoA angle in $\theta$ and $\phi$ can further be retrieved by the peak-finding algorithm^18^. The system-level blocks for DoA estimation are shown in Fig. 1 as a part of the baseband processing unit. The DoA estimation depends upon the current cavity state, defining the field patterns on the characteristic plan (or measurement modes) for discrete frequencies within $\omega$. This is similar to the measurement modes in microwave imaging^12,13,15,16^ in which for the same cavity state, when the driving frequency of the cavity is varied, the radiation masks changes. This leads to a diverse set of measurement modes by moving along the frequency axis (for example in Fig. 3). The lens structure enhances the gain of the sidelobes probing the FoV; hence, sharpening the masks further and reducing the overlap between masks in neighbouring frequencies.

Now let us examine when the SADEA-I-based optimization process is initialized and the lens-loaded cavity static state is updated via the rotation of the stepper motor shaft for the first design from the initial database of SADEA-I. The set of modes generated by the previous state of the lens-loaded cavity are no longer valid, and a new set of modes are generated, given the frequency-diverse functionality of high-Q chaotic cavity. Hence, the previous $E(r,\omega ,1)$ and estimated $P_{est,M,1}$ are also no longer valid; however, they are buffered to be used by the surrogate model to evaluated $E(r,\omega ,2)$ and $P_{est,M,2}$ for the updated state of the lens-loaded cavity (i.e., the subsequent designs generated by SADEA and each new design is numbered in 3rd subscript), here, represented as $k\in [0^\circ , \, 360^\circ ]$ i.e., updated cavity state number. Note that only one (the best) state of the cavity is used for DoA estimation. Also, consider the number of resonances over a specific bandwidth, $N_R$, from the full-wave EM simulation of each design (i.e., each static state of the lens-loaded cavity for a single *E*). These designs (i.e., *k*) and the associated $N_R$ are used for a SADEA-I-based optimization of the lens-loaded cavity aperture for the first time in this paper, as described in the next section.

## Example and discussion

Considering the lens-loaded cavity in Fig. 2a described in the previous section as having a mode-mixing scatterer whose orientation defines or determines the state of the chaotic cavity. To have the best number of practical modes to ensure the best frequency-diverse performance of the chaotic cavity, the orientation of the metallic scatter (*k*) is optimized by SADEA-I using the following goal:

13 $$\begin{aligned} \text{ maximize } \quad (N_{R}) \quad 27\,{\text {GHz }} {\text { to }} 29\,{\text {GHz}} \end{aligned}$$

**Figure 5:**
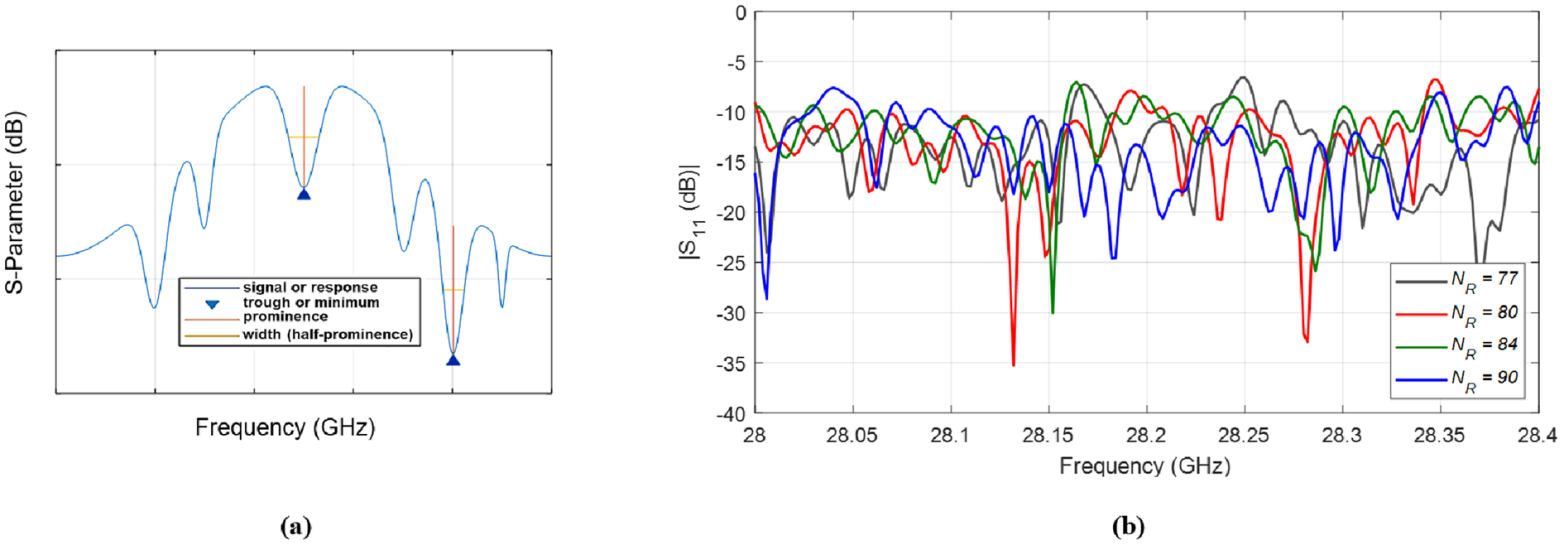
(**a**) Prominence and width of a resonance for a given frequency response. (**b**) Comparison between the frequency responses of selected designs generated during the SADEA-I-based optimization.

Using a single rotating frame of reference as illustrated in Fig. 2, the search range for *k* is defined as 0$^{\circ }$ to 360$^{\circ }$. In other words, the optimization is over a continuous space. For a given *k* such as $k_{i}$ in the optimization process, as earlier discussed and illustrated in Fig. 5a, $N_R$ is defined as the total number of resonances in the frequency response for $k_{i}$. The condition used to judge whether a resonance exists is if the corresponding prominence is not smaller than 1 dB in terms of the S-Parameter values over a frequency range not greater than 0.5 GHz in the given bandwidth. The computing budget used for the SADEA-I is set as 200 full-wave EM simulations and the convergence criterion used is that if $N_R$ does change or improve after 20 full-wave EM simulations.

SADEA-I is implemented in MATLAB^54^ and the EM simulator is CST Microwave Studio (CST-MWS). The working environment is a Red Hat Enterprise Linux Server 7.6 (Maipo 7.6 64-bit) where all EM simulations were carried out using CST-MWS distributed computing feature with $2 \times 18$ Core 3.30 GHz processor and 2 Tesla M60 accelerator devices with 755.6 GB RAM. The simulation model was discretized using a mesh density of 3 cells per wavelength to have around 8.5 million mesh cells in total, and each full-wave EM simulation costs about 70 to 80 minutes on average.

**Figure 6:**
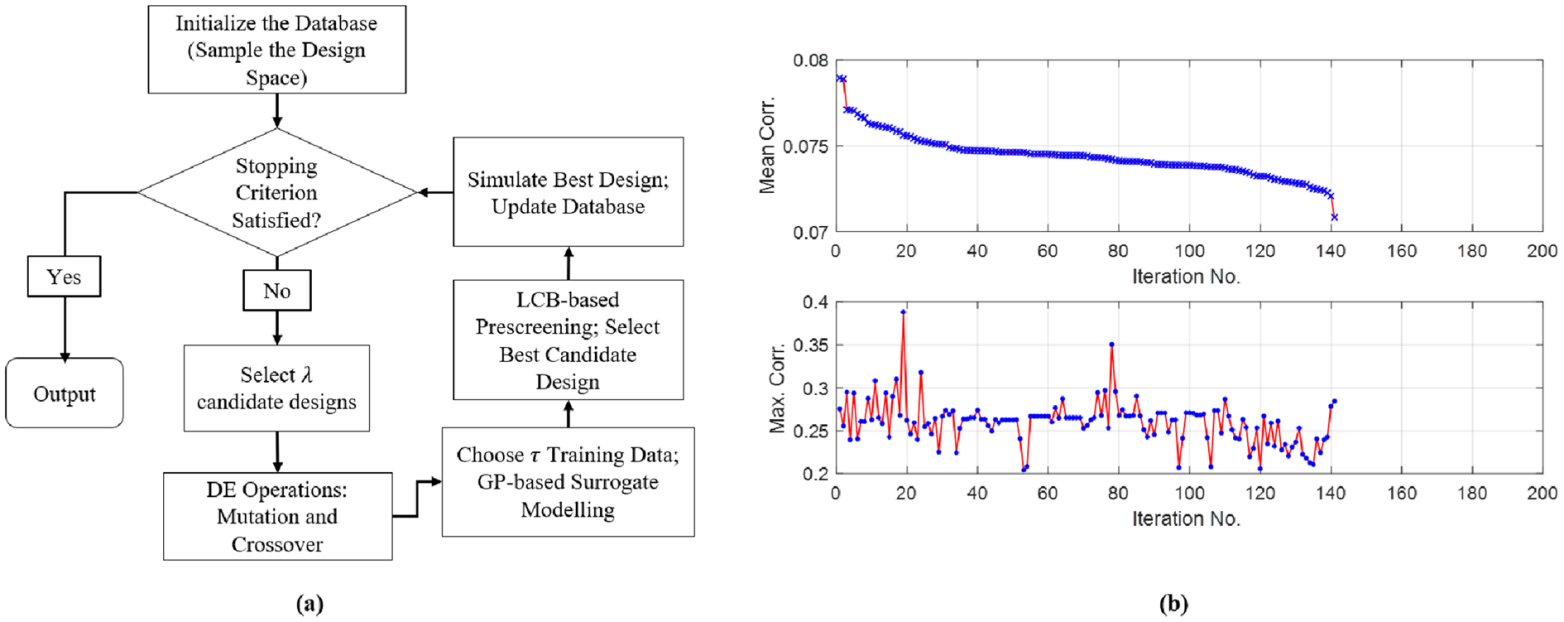
(**a**) Flow diagram of SADEA-I. (**b**) Comparison between mean and maximum correlation coefficients when mean correlation coefficient against each iteration is plotted in descending order.

For clarity, the increasing number of resonances (i.e., $N_R$) during the optimization process is shown in Fig. 5b within the frequency range of 28–28.4 GHz for four randomly selected designs. It can be observed that the return loss response of the chaotic cavity gets updated for every design generated in the optimization process, confirming the frequency-diverse operation. The flow diagram of how SADEA-I worked for aperture optimization is shown in Fig. 6a. Following the stopping criterion, after 33 full-wave EM simulations using 660 surrogate models (calculated by $\alpha$ $\times$ number of optimization goals and/or targets $\times$ the total number of full-wave EM simulations used), SADEA-I converged to obtain the optimized design: $k=280.11^{\circ }$ with $N_R=94$.

**Figure 7:**
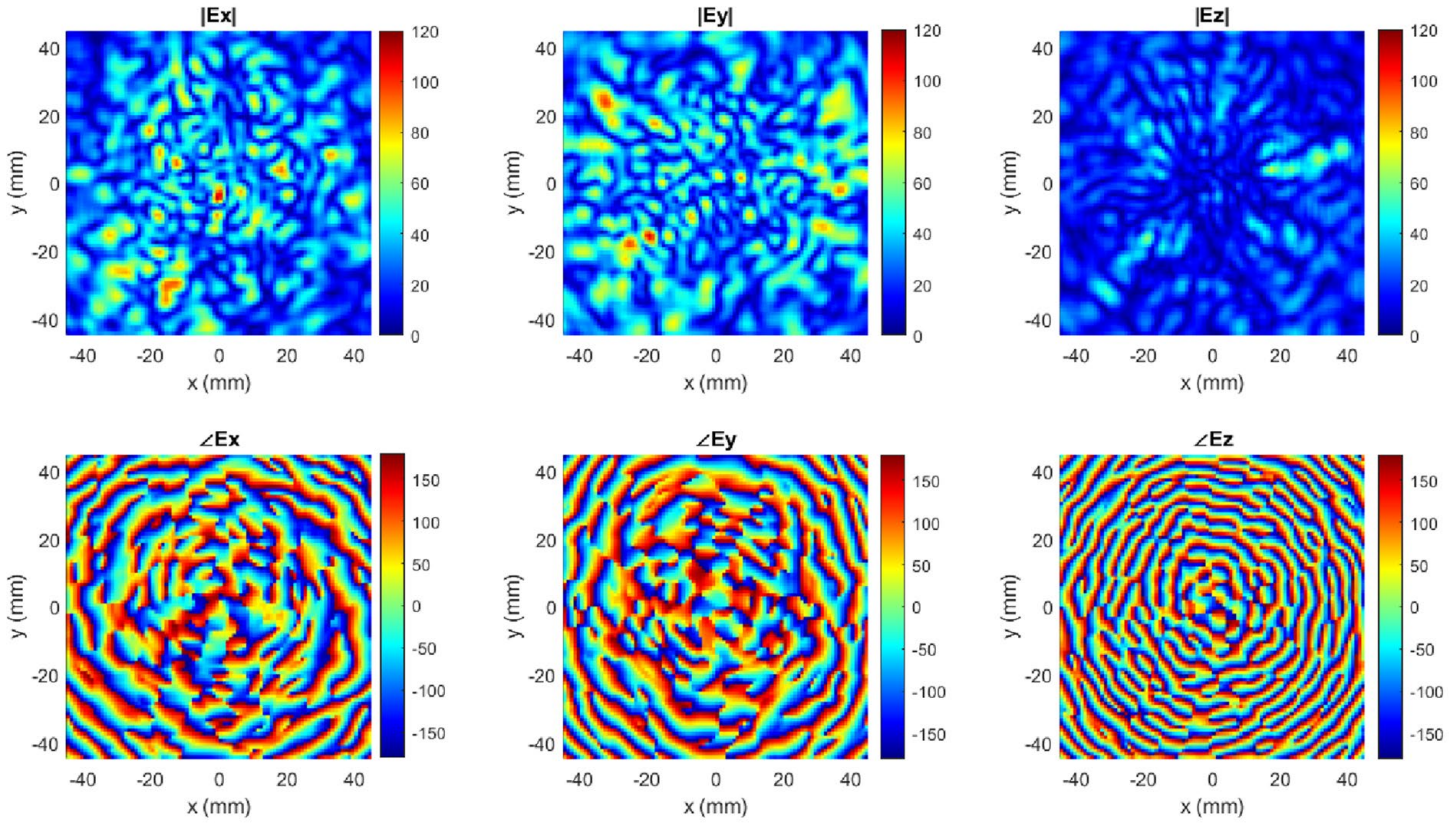
Simulated *x*, *y* and *z* components of *E*-field (V/m) in terms of magnitude and phase contour plots.

In addition to the increase in $N_R$, the spatial quasi-randomness of the radiated field and its low correlation with the fields of the neighboring modes helps in conditioning a frequency-diverse cavity for enhanced DoA estimation accuracy^18,19^. Based on this principle, it is assumed that minimizing the correlation between aperture radiated fields projected on a characterization plane for neighboring modes against a single lens-loaded aperture state *k* can provide even a refined solution. To take this into account, the following optimization criterion is proposed to determine the best value of *k*:

14 $$\begin{aligned} \text{ minimize } \;\;\; (C_{AP}) + \text{ maximize } \;\;\; (N_R) \;\;\; 27{-}29\,{\text {GHz}} \end{aligned}$$

where $C_{AP}$ is the mean correlation between all the radiation modes for single lens-loaded aperture state *k*, defined as follows:

15 $$\begin{aligned} C_{AP} = \frac{\sum _{i=1}^{N_R-1} Corr\left( E(\omega _i, k),E(\omega _{i+1}, k)\right) }{N_R}. \end{aligned}$$

It is expected that reduced correlation between neighboring modes will enhance the amplitude and phase of the impinging signal or channel state parameters in a given FoV. Note that in (14), minimizing the maximum correlation can also be used as a criterion calculated from all the radiation modes at a given state *k* instead of calculating mean correlation to optimize a lens-loaded dynamic aperture.

In our example, since we already have an optimized solution $k=280.11^{\circ }$, a simplified optimization criteria below is used to get to a final solution:

16 $$\begin{aligned} \begin{array}{ll} \text{ minimize } \;\;\;\; (C_{AP}) \;\;\; 27{-}29\,{\text {GHz}}\\ s.t. \\ \quad N_{R} \ge 90 \\ \end{array} \end{aligned}$$

Note that the threshold of 90 used for the constraint imposed on $N_R$ has been informed from the result of the previous optimization carried out and the search range for *k* is the same as stated for the previous optimization (i.e., this optimization is also over a continuous space). The computing budget and convergence criterion are exactly the same as stated for the previous optimization. To better understand the characterization plane fields of neighboring modes factored as the correlation between neighboring modes in the optimization process, the simulated components of the fields on the characterization plane ($E(\omega )$) for $k=280.11^{\circ }$ at 28.1 GHz are shown in Fig. 7.

The convergence trend for the minimization of the objective function (i.e., $C_{AP}$) is shown in Fig. 6b. Considering the optimization goal stated in (16), after 142 full-wave EM simulations using 5,680 surrogate models (calculated by $\alpha$ $\times$ number of optimization goals and/or targets $\times$ the total number of full-wave EM simulations used), the final design obtained is $k=3.665^{\circ }$, having $C_{AP}=0.070832935$ and $N_R=90$. Moreover, it can be seen that while the ML-assisted optimization process tried to reduce the mean correlation, the maximum correlation did not follow the same trend, until beyond iteration No. 120.

**Figure 8:**
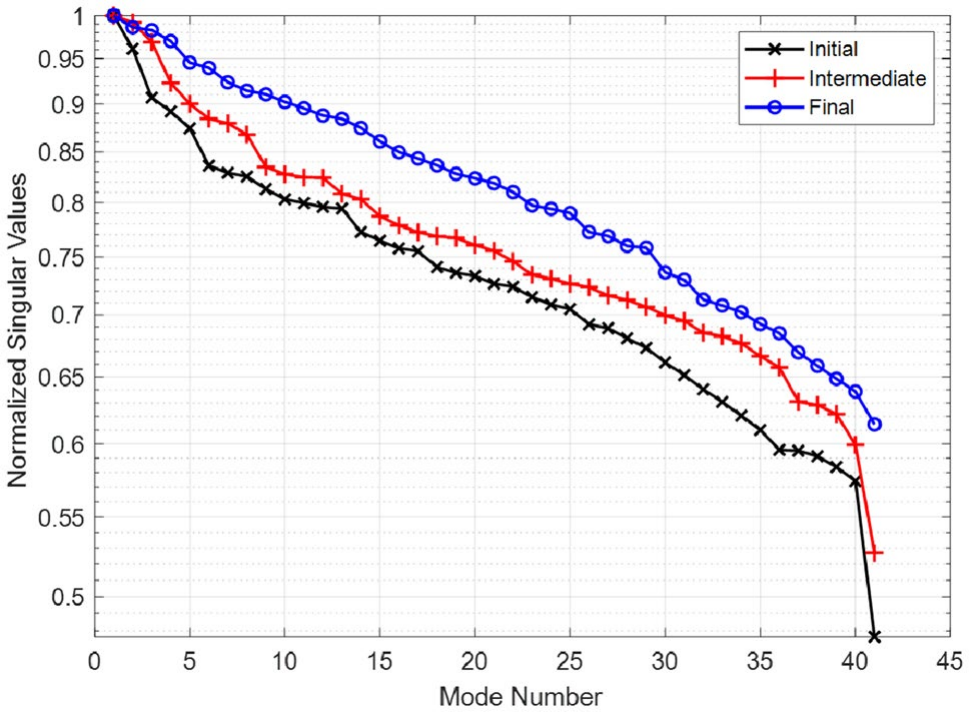
Singular value comparison between initial, intermediate and final optimized states of the lens-loaded cavity.

A quantitative analysis of the orthogonality of the modes radiated by the chaotic cavity can be carried out in the context of a singular value decomposition (SVD) study. In Fig. 8, we present the singular values of the cavity radiated modes for the initial, intermediate, and final optimized configurations. From Fig. 8, the decay slope of the SVD pattern for the initial cavity design is deduced to be 0.52/41 modes (or − 6.4 dB/41 modes), whereas the SVD decay slopes for the intermediate and final optimized designs are deduced to be 0.47/41 modes (or − 5.56 dB/41 modes) and 0.38/41 modes (or − 4.23 dB/41 modes), respectively. The decay slope of the SVD pattern is an important metric because this slope governs the correlation between the antenna radiated modes^13–15^. In other words, higher SVD decay slopes correspond to reduced orthogonality between the radiated modes, reducing the information content captured by each mode. In contrast, a smaller SVD decay slope suggests higher orthogonality of the radiated modes, increasing the information content captured by each mode.

### DoA estimation results

**Figure 9:**
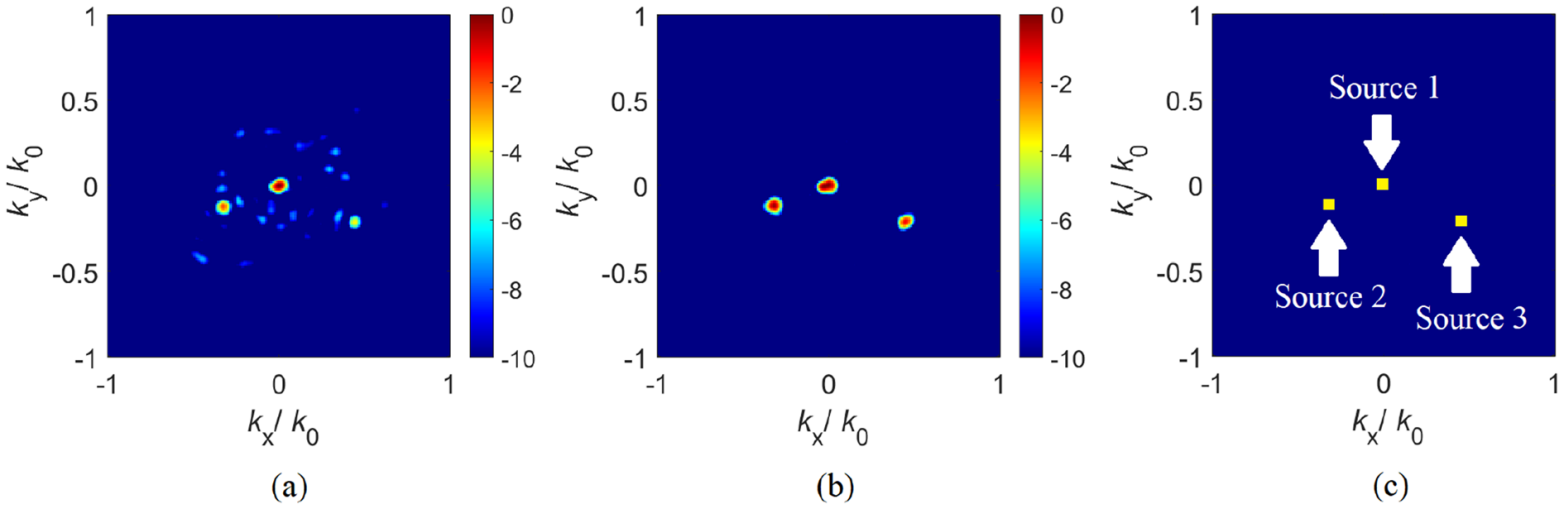
Reconstructed DoA patterns using (**a**) initial cavity configuration (**b**) optimized (final) cavity configuration. Original distribution of sources (ground truth) is shown in (**c**). Colorbar: dB.

Following the optimization of the chaotic cavity, a performance analysis of the cavity can be performed by considering an example DoA estimation scenario. For this study, we define an arbitrarily selected number of far-field sources that are incident on the aperture of the cavity at ($\theta _1=0^\circ ,\,\phi _1=0^\circ$), ($\theta _2=-20^\circ ,\,\phi _2= 20^\circ$) and ($\theta _3=30^\circ ,\,\phi _3=-25^\circ$) respectively. To retrieve the DoA pattern, we use the initial (before training) and final (after training) set of modes radiated from the cavity. The DoA retrieval is accomplished by solving the least-squares problem of Eq. (12) and the reconstructed DoA patterns are shown in Fig. 9.

**Figure 10:**
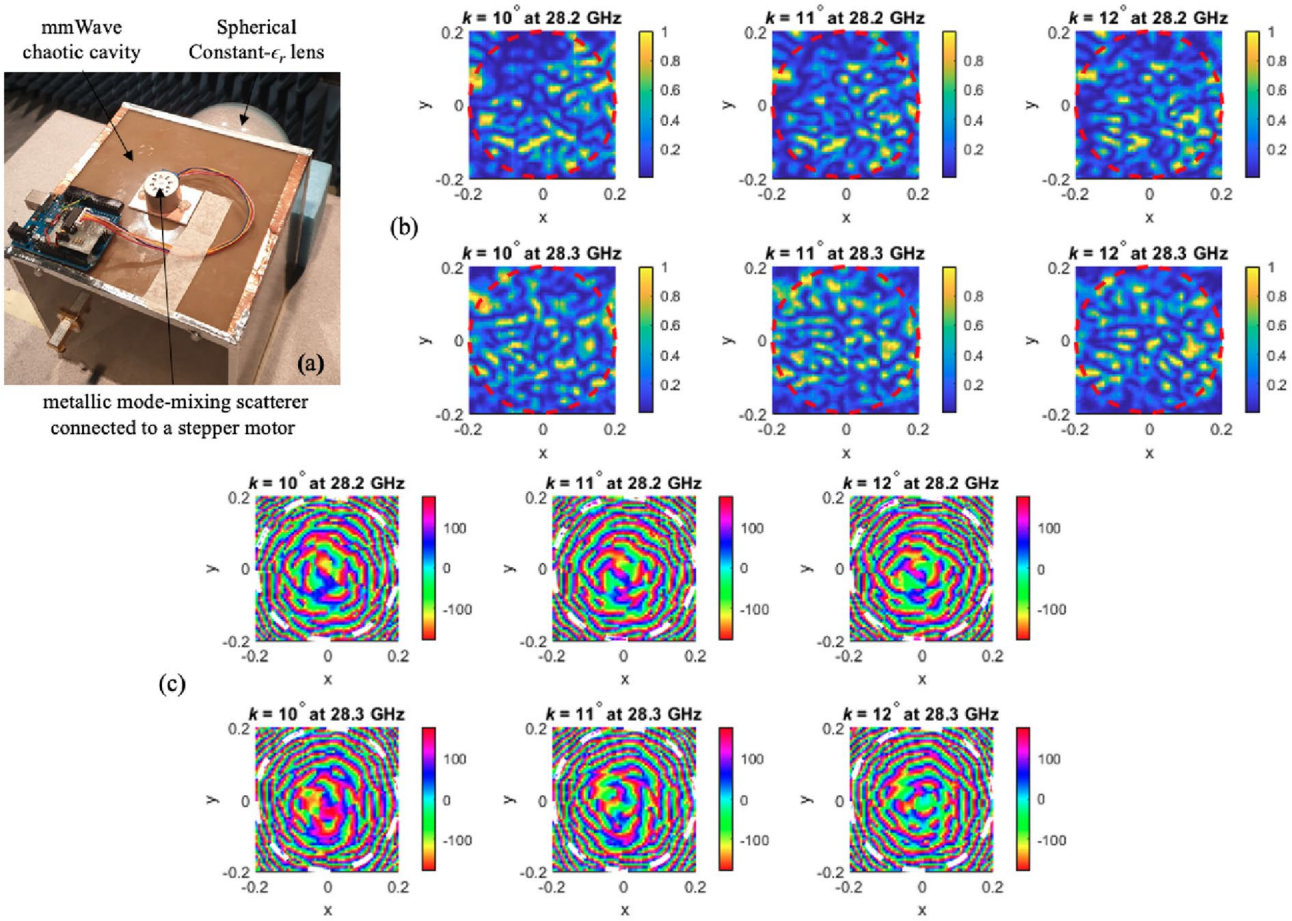
(**a**) Stepper motor connected to the lens-loaded cavity. Measured *y*-components of *E*-field in terms of (**b**) normalized magnitude (V/m) and (**c**) phase (degree) contour plots.

As can be seen in Fig. 9, the DoA pattern reconstructed using the optimized cavity modes exhibits better fidelity. Whereas all far-field sources are clearly distinguished in the retrieved DoA pattern reconstructed using the optimized (final) cavity configuration in Fig. 9b, the DoA pattern reconstructed using the initial (non-optimized) cavity retrieved only some of the sources and with a substantially reduced accuracy. A quantitative assessment of the DoA estimations demonstrated in Fig. 9 is provided in Table 1.

**Table 1:** Analysis of the DoA reconstruction fidelity. The original (ground truth) DoA values are compared to the estimated DoA values reconstructed using initial and final modes.

Source	DoA (ground truth)	DoA (estimated)—initial	DoA (estimated)—final
Source 1	$(\theta _{1}=0^\circ ,\,\phi _{1}=0^\circ )$	$(\theta _{1,est}=0^\circ ,\,\phi _{1,est}=0^\circ )$	$(\theta _{1,est}=0^\circ ,\,\phi _{1,est}=0^\circ )$
Source 2	$(\theta _{2}=-20^\circ ,\,\phi _{2}=20^\circ )$	$(\theta _{2,est}=-22.6^\circ ,\,\phi _{2,est}=21.4^\circ )$	$(\theta _{2,est}=-20.1^\circ ,\,\phi _{2,est}=20.1^\circ )$
Source 3	$(\theta _{3}=30^\circ ,\,\phi _{3}=-25^\circ )$	$(\theta _{3,est}=\text {N/A},\,\phi _{3,est}=\text {N/A})$	$(\theta _{3,est}=30^\circ ,\,\phi _{3,est}=-25.4^\circ )$

Analyzing Table 1, it is evident that the DoA estimation obtained with the initial cavity configuration exhibits larger discrepancies between the estimated and original DoA values, and also fails to retrieve source 3. On the contrary, the DoA values estimated using the final, optimized cavity configuration are in good agreement with the original DoA values. In addition, all far-field sources, in this case, are clearly identified.

### SVD results

The advantage of the optimized chaotic cavity can be further seen by evaluating the ratio of the largest values to the smallest singular values in the SVD pattern, known as the condition number (CN)^15^. In Eq. (11), an increasing CN for the E-matrix calculated from its SVD analysis would suggest an ill-conditioned problem for recovering $P_{est}$, whereas an E-matrix with CN closer to unity would suggest a better-conditioned problem. In this context, the CN for the optimized design is calculated as $CN_{1}=1.61$ whereas for the intermediate and initial designs, it is calculated as $CN_{2}=1.92$ and $CN_{3}=2.13$, respectively. In other words, the CN of the optimized design is 25% smaller than the CN of the initial design.

### Dynamic aperture validation results

A mode-mixing scatterer is implemented on the same hardware shown in^19,20^. The lens-loaded cavity structure with the scatterer is connected to the stepper motor in a similar manner as shown in the simulated model in Fig. 2. The dynamic aperture is then placed in the near-field anechoic chamber where co-polarized complex *E*-field is measured. To test the sensitivity of the cavity, the mode-mixing scatterer is rotated just by $1^\circ$ to create three static cavity states, while the resultant fields against each state are recorded at an observation plane, shown in Fig. 10. The radiation modes can be observed to be updated when comparing the contour plots against $k = 10^\circ$, $11^\circ$, and $12^\circ$, and this is true for both the magnitude (Fig. 10b) as well as the phase plots (Fig. 10c). This confirms the simulated predictions and the validity of the dynamic aperture operation discussed in section “System model and methods”.

## Conclusion

In this paper, it has been shown that a mechanically controlled mode-mixing scatterer can dynamically update the state of the lens-loaded aperture, optimized by the SADEA-I method, to provide a state best suited for improved DoA estimation accuracy. To quantitatively analyze the achievable improvement, we first optimized the aperture to maximize the number of radiation modes, and afterward optimized it to simultaneously have a large number of radiation modes as well as a reduced amount of correlation between the radiation modes at adjacent frequency points. The optimization process shown in this work is purely simulation-driven, while it verifies the functionality of our unique enabling technology of real-time lens-loaded cavity optimization in practical channels. It is shown that a mechanical rotation of the mode-mixing scatterer inside the lens-loaded cavity can produce a unique set of frequency-diverse modes and radiation masks. If this rotation is optimized using SADEA-I based on a given criterion, it can improve the dynamic aperture conditioning to enable accurate DoA estimation verified in this paper by full-wave EM simulations campaign. To quantify the benefits of the proposed technique, we show the singular value decomposition spectrum against the initial, intermediate, and final state of the lens-loaded cavity, revealing a 25$\%$ reduction in the CN when comparing the initial with final state. Finally, DoA estimation patterns using initial and final cavity modes are compared with the ground truth to verify the validity of the dynamic aperture optimisation method. Future works include investigation of practical mode-mixing mechanism in a lens-loaded cavity hardware and practical verification of dynamic aperture optimization using the SADEA-I method.

## Data Availability

All data is provided in full in the results section of this paper.

## References

[1] Gao F, Gershman AB (2005). A generalized ESPRIT approach to direction-of-arrival estimation. IEEE Signal Process. Lett..

[2] Zhang X, Huang Y, Chen C, Li J, Xu D (2012). Reduced-complexity capon for direction of arrival estimation in a monostatic multiple-input multiple-output radar. IET Radar Sonar Navig..

[3] Sim H, Lee S, Kang S, Kim S-C (2019). Enhanced DOA estimation using linearly predicted array expansion for automotive radar systems. IEEE Access..

[4] Mohanna M, Rabeh ML, Zieur EM, Hekala S (2013). Optimization of music algorithm for angle of arrival estimation in wireless communications. NRIAG J. Astron. Geophys..

[5] Kintz AL, Gupta IJ (2016). A modified music algorithm for direction of arrival estimation in the presence of antenna array manifold mismatch. IEEE Trans. Antennas Propag..

[6] Aslan Y (2018). Thermal-aware synthesis of 5g base station antenna arrays: An overview and a sparsity-based approach. IEEE Access.

[7] Chiu, C.-P. Heat sink for 5g massive antenna array and methods of assembling same (2019). US Patent 10,320,051.

[8] Vlachos, E., Thompson, J., Abbasi, M. A. B., Fusco, V. F. & Matthaiou, M. Robust estimator for lens-based hybrid MIMO with low-resolution sampling. In *2019 IEEE 20th Int. Workshop on Signal Process. Advances in Wireless Commun. (SPAWC)*, 1–5 (IEEE, 2019).

[9] Giordani M, Polese M, Roy A, Castor D, Zorzi M (2018). A tutorial on beam management for 3GPP NR at mmWave frequencies. IEEE Commun. Surveys Tuts..

[10] Shu F (2018). Low-complexity and high-resolution DOA estimation for hybrid analog and digital massive MIMO receive array. IEEE Trans. Commun..

[11] Abbasi MAB, Fusco V, Zelenchuk DE (2018). Compressive sensing multiplicative antenna array. IEEE Trans. Antennas Propag..

[12] Zhao M (2019). Frequency-diverse bunching metamaterial antenna for coincidence imaging. Materials.

[13] Yurduseven O (2017). Computational microwave imaging using 3D printed conductive polymer frequency-diverse metasurface antennas. IET Microw. Antennas Propag..

[14] Fromenteze T (2015). Computational imaging using a mode-mixing cavity at microwave frequencies. Appl. Phys. Lett..

[15] Yurduseven O, Abbasi MAB, Fromenteze T, Fusco V (2020). Lens-loaded coded aperture with increased information capacity for computational microwave imaging. Remote Sens..

[16] Yurduseven O (2015). Resolution of the frequency diverse metamaterial aperture imager. Prog. Electromagn. Res..

[17] Hoang TV (2021). Spatial diversity improvement in frequency-diverse computational imaging with a multi-port antenna. Results Phys..

[18] Yurduseven O, Abbasi MAB, Fromenteze T, Fusco V (2019). Frequency-diverse computational direction of arrival estimation technique. Sci. Rep..

[19] Abbasi M, Fusco V, Yurduseven O, Fromenteze T (2020). Frequency-diverse multimode millimetre-wave constante lens-loaded cavity. Sci. Rep..

[20] Abbasi, M. A. B., Fusco, V. F. & Yurduseven, O. Millimeter-wave channel sounding technique using oversized lens-loaded cavity. In *2021 15th European Conference on Antennas and Propagation (EuCAP)*, 1–3 (IEEE, 2021).

[21] Liu B (2014). An efficient method for antenna design optimization based on evolutionary computation and machine learning techniques. IEEE Trans. Antennas Propag..

[22] Liu B, Koziel S, Ali N (2017). Sadea-II: A generalized method for efficient global optimization of antenna design. J. Comput. Design Eng..

[23] Liu B, Akinsolu MO, Ali N, Abd-Alhameed R (2019). Efficient global optimisation of microwave antennas based on a parallel surrogate model-assisted evolutionary algorithm. IET Microw. Antennas Propag..

[24] Akinsolu MO (2019). A parallel surrogate model assisted evolutionary algorithm for electromagnetic design optimization. IEEE Trans. Emerg. Top. Comput. Intell..

[25] Liu B (2021). An efficient method for complex antenna design based on a self adaptive surrogate model-assisted optimization technique. IEEE Trans. Antennas Propag..

[26] Grout V (2019). Software solutions for antenna design exploration: A comparison of packages, tools, techniques, and algorithms for various design challenges. IEEE Antennas Propag. Mag..

[27] Liu B, Zhang Q, Gielen GGE (2014). A gaussian process surrogate model assisted evolutionary algorithm for medium scale expensive optimization problems. IEEE Trans. Evol. Comput..

[28] Abbasi MAB, Fusco VF, Tataria H, Matthaiou M (2019). Constant-$$\epsilon _r$$ lens beamformer for low-complexity millimeter-wave hybrid MIMO. IEEE Trans. Microw. Theory Tech..

[29] Chen X, Gu C, Zhang Y, Mittra R (2018). Analysis of partial geometry modification problems using the partitioned-inverse formula and Sherman–Morrison–Woodbury formula-based method. IEEE Trans. Antennas Propag..

[30] Chen X, Liu X, Gu C (2020). Efficient calculation of interior scattering from cavities with small modifications. Electron. Lett..

[31] Koziel, S. & Ogurtsov, S. *Antenna Design by Simulation-Driven Optimization* (Springer, 2014).

[32] Werner, D. H., Gregory, M. D., Jiang, Z. H. & Brocker, D. Optimization methods in antenna engineering. In *Handbook of Antenna Technologies*, 321–376 (Springer, 2016).

[33] Lazaridis PI (2016). Comparison of evolutionary algorithms for LPDA antenna optimization. Radio Sci..

[34] Li X, Luk KM (2020). The grey wolf optimizer and its applications in electromagnetics. IEEE Trans. Antennas Propag..

[35] Al-Azza AA, Al-Jodah AA, Harackiewicz FJ (2016). Spider monkey optimization: A novel technique for antenna optimization. IEEE Antennas Wirel. Propag. Lett..

[36] Akinsolu, M. O., Mistry, K. K., Liu, B., Lazaridis, P. I. & Excell, P. Machine learning-assisted antenna design optimization: A review and the state-of-the-art. In *2020 14th European Conference on Antennas and Propagation (EuCAP)*, 1–5. 10.23919/EuCAP48036.2020.9135936 (2020).

[37] Wu, Q., Cao, Y., Wang, H. & Hong, W. Machine-learning-assisted optimization and its application to antenna designs: Opportunities and challenges. *China Commun.* **17**, 152–164. 10.23919/JCC.2020.04.014 (2020).

[38] El Misilmani HM, Naous T, Al Khatib SK (2020). A review on the design and optimization of antennas using machine learning algorithms and techniques. Int. J. RF Microw. Comput. Aided Eng..

[39] Zhang Z, Chen HC, Cheng QS (2021). Surrogate-assisted quasi-newton enhanced global optimization of antennas based on a heuristic hypersphere sampling. IEEE Trans. Antennas Propag..

[40] Koziel S, Bandler JW, Cheng QS (2010). Robust trust-region space-mapping algorithms for microwave design optimization. IEEE Trans. Microw. Theory Tech..

[41] Koziel S, Pietrenko-Dabrowska A (2019). Reduced-cost electromagnetic-driven optimisation of antenna structures by means of trust-region gradient-search with sparse jacobian updates. IET Microw. Antennas Propag..

[42] Bekasiewicz A, Koziel S (2019). Reliable multistage optimization of antennas for multiple performance figures in highly dimensional parameter spaces. IEEE Antennas Wirel. Propag. Lett..

[43] Pietrenko-Dabrowska A, Koziel S (2020). Antenna modeling using variable-fidelity EM simulations and constrained co-kriging. IEEE Access.

[44] Song Y, Cheng QS, Koziel S (2019). Multi-fidelity local surrogate model for computationally efficient microwave component design optimization. Sensors.

[45] Koziel, S. & Pietrenko-Dabrowska, A. Accelerated gradient-based optimization of antenna structures using multi-fidelity simulations and convergence-based model management scheme. *IEEE Trans. Antennas Propag.* 1. 10.1109/TAP.2021.3083742 (2021).

[46] Santner, T. J., Williams, B. J., Notz, W. I. & Williams, B. J. *The Design and Analysis of Computer Experiments*, Vol. 1 (Springer, 2003).

[47] Rasmussen, C. Gaussian processes in machine learning. *Advanced Lectures on Machine Learning*, 63–71 (2004).

[48] Emmerich M, Giannakoglou K, Naujoks B (2006). Single- and multiobjective evolutionary optimization assisted by gaussian random field metamodels. IEEE Trans. Evol. Comput..

[49] Dennis J, Torczon V (1997). Managing approximation models in optimization. Multidiscip. Design Optim. State-of-the-art.

[50] Storn R, Price K (1997). Differential evolution-a simple and efficient heuristic for global optimization over continuous spaces. J. Global Optim..

[51] Stein, M. Large sample properties of simulations using Latin hypercube sampling. *Technometrics* 143–151 (1987).

[52] Yurduseven O, Abbasi MAB, Fromenteze T, Fusco V (2019). Frequency-diverse computational direction of arrival estimation technique. Sci. Rep..

[53] Sharma, R., Fusco, V., Yurduseven, O. *et al.* Single-pixel compressive direction of arrival estimation using programmable metasurface apertures. In *Passive and Active Millimeter-Wave Imaging XXIV*, Vol. 11745, 117450B (International Society for Optics and Photonics, 2021).

[54] Liu B (2017). Gui design exploration software for microwave antennas. J. Comput. Design Eng..

